# Abdominal compartment syndrome following chemotherapy-induced gastrointestinal mucositis: a case report

**DOI:** 10.3389/fmed.2026.1718333

**Published:** 2026-01-16

**Authors:** Tian-le Li, Meng Li, Xiao-ming Yang, Jin Wei, Lei Huang

**Affiliations:** 1 Heart Center, Central Hospital, Tianjin University/Tianjin Third Central Hospital, Tianjin, China; 2 Tianjin Key Laboratory of Extracorporeal Life Support for Critical Diseases, Tianjin Artificial Cell Engineering Technology Research Center, Tianjin Institute of Hepatobiliary Disease, Nankai University Affinity the Third Central Hospital, Tianjin, China; 3 Department of Critical Care Medicine, Central Hospital, Tianjin University/Tianjin Third Central Hospital, Tianjin, China; 4 Department of Emergency, Central Hospital, Tianjin University/Tianjin Third Central Hospital, Tianjin, China; 5 Ward General Practice, Central Hospital, Tianjin University/Tianjin Third Central Hospital, Tianjin, China

**Keywords:** abdominal compartment syndrome, case report, chemotherapy, gastrointestinal mucositis, sepsis

## Abstract

**Background:**

Abdominal compartment syndrome (ACS) is a life-threatening condition typically associated with trauma, major surgery, or acute abdominal pathology. Its occurrence in oncology patients, particularly as a complication of chemotherapy-induced gastrointestinal mucositis, is exceedingly rare. This report presents a unique case of ACS triggered by mucosal injury following chemotherapy, complicated by septic shock and multiorgan failure, in a patient with lung cancer.

**Case presentation:**

A 64-year-old male with lung cancer underwent platinum-etoposide chemotherapy. Following his fourth cycle, he developed bowel dysmotility and was administered a laxative, which precipitated profuse watery diarrhea. Within hours, he experienced progressive abdominal distension, nausea, vomiting, and hemodynamic instability. Laboratory tests revealed severe metabolic acidosis, hyperlactatemia, and pancytopenia. Imaging showed massive gastrointestinal fluid retention and elevated intra-abdominal pressure (IAP). Blood cultures confirmed ESBL-producing *Escherichia coli* bacteremia. The patient was admitted to the intensive care unit and received mechanical ventilation, vasopressors, continuous renal replacement therapy, gastrointestinal decompression, and broad-spectrum antibiotics. IAP peaked at 28 mmHg, fulfilling criteria for ACS. With multidisciplinary management, including PiCCO-guided hemodynamic optimization and targeted antimicrobial therapy, the patient’s condition gradually improved. He was extubated on day 17 and discharged from the intensive care unit on day 19.

**Conclusion:**

This case illustrates a rare but critical pathway from chemotherapy-induced mucosal injury to ACS, mediated by enterogenic sepsis and systemic inflammation. Early recognition, continuous IAP monitoring, and aggressive supportive care are essential for survival. Clinicians should maintain a high index of suspicion for ACS in immunocompromised oncology patients presenting with abdominal symptoms and septic physiology.

## Introduction

Abdominal compartment syndrome (ACS) is a critical condition defined by sustained intra-abdominal pressure (IAP) ≥20 mmHg associated with new-onset organ dysfunction (1). It is most encountered in the context of trauma, major abdominal surgery, severe pancreatitis, or massive fluid resuscitation (2–4). In contrast, ACS arising in oncology patients—particularly as a downstream complication of chemotherapy—is exceedingly rare and poorly characterized in the literature.

Chemotherapy-induced gastrointestinal mucositis is a well-recognized adverse effect of cytotoxic regimens, especially those involving platinum-based agents and etoposide (5). This mucosal injury disrupts epithelial integrity, impairs barrier function, and predisposes immunocompromised patients to bacterial translocation and systemic infection (6). In severe cases, this cascade can evolve into septic shock, capillary leak syndrome, and intra-abdominal fluid accumulation, setting the stage for escalating IAP and ACS (7). However, the full trajectory from mucositis to ACS has not been comprehensively documented in prior case reports or cohort studies.

This case report presents a rare and instructive example of ACS developing in a patient with lung cancer following combination chemotherapy. The patient’s clinical course was marked by rapid deterioration: chemotherapy-induced mucosal injury, laxative-triggered diarrhea, enterogenic sepsis with ESBL-producing *Escherichia coli*, multiorgan failure, and sustained IAP elevation requiring intensive care interventions. The diagnosis of ACS was confirmed through continuous IAP monitoring and correlated with dynamic organ dysfunction, including acute kidney injury, septic cardiomyopathy, and respiratory failure.

Beyond its rarity, this case offers several contributions to the existing body of knowledge. First, it highlights the importance of recognizing ACS in non-surgical, immunocompromised populations—a context often overlooked in critical care algorithms. Second, it underscores the diagnostic value of early IAP surveillance in patients with abdominal distension and septic physiology, even in the absence of overt peritonitis or surgical pathology. Third, it demonstrates that ACS in oncology patients can be successfully managed with a nonoperative, protocolized approach involving gastrointestinal decompression, PiCCO-guided fluid stewardship, and continuous renal replacement therapy.

To our knowledge, this is one of the first case reports to delineate the full pathophysiological arc from chemotherapy-induced mucositis to ACS, supported by serial laboratory, imaging, and hemodynamic data. It fills a gap in the literature by contextualizing ACS within the framework of oncologic critical care and offers practical insights for clinicians managing similar high-risk scenarios.

## Case presentation

A 64-year-old male with hypertension, chronic tobacco and alcohol use, and biopsy-confirmed small cell neuroendocrine carcinoma of the right upper-lobe mediastinal region—diagnosed by bronchoscopy with endobronchial ultrasound-guided biopsy and treated with platinum-etoposide chemotherapy—had completed his fourth chemotherapy cycle the previous day. Later that day, he developed constipation and took a laxative (Cannabis seed capsule), followed by montmorillonite powder after the onset of watery diarrhea. His abdominal bloating and chest discomfort progressively worsened, prompting presentation to the emergency department.

On arrival, the patient was alert and oriented. Vital signs were: temperature 36.5 °C, heart rate 167 bpm, blood pressure 108/67 mmHg, respiratory rate 20 breaths/min, and peripheral oxygen saturation 98%. Physical examination revealed a distended abdomen with mild tenderness in the epigastric and lower quadrants, coarse breath sounds bilaterally without rales, and no peripheral edema. Electrocardiography showed supraventricular tachycardia without ST-T changes, which was managed with intravenous verapamil and beta-blockers.

**Figure 1:**
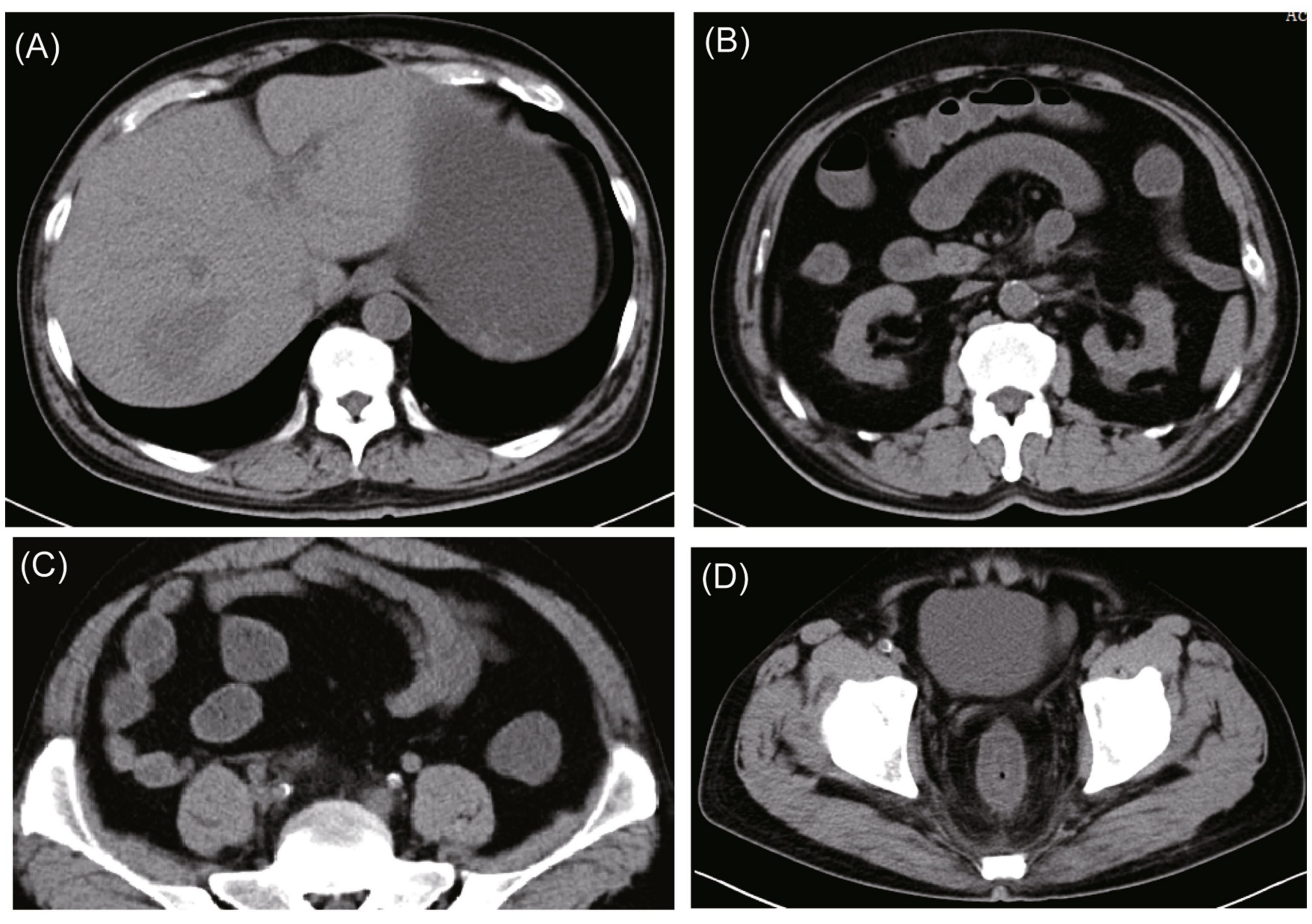
Abdomen and pelvic CT scan on hospital day 1. **(A)** The liver shows a smooth contour with well-defined margins and proportionate lobar anatomy. A patchy hypodense lesion is observed in the posterior segment of the right hepatic lobe, measuring approximately 5.9 × 4.8 cm, with ill-defined borders. **(B,C)** The bowel loops demonstrate marked gaseous and fluid distension, consistent with impaired motility. **(D)** The rectal wall appears thickened, with increased density of the surrounding mesenteric fat, showing stranding and streak-like opacities suggestive of inflammatory changes. No significantly enlarged lymph nodes are identified in the abdominal cavity, retroperitoneum, pelvic walls, or bilateral inguinal regions.

Initial laboratory tests revealed mildly elevated creatinine (222 μmol/L), urea (18.32 mmol/L), and uric acid (557 μmol/L), with normal liver enzymes (AST 32.0 U/L, ALT 26.0 U/L), electrolytes, and glucose (6.04 mmol/L). Cardiac biomarkers (cTnI, CK-MB, myoglobin), BNP, and amylase were within normal limits. D-dimer was markedly elevated at 2.406 mg/L (reference <0.3 mg/L). Complete blood count showed WBC 6.21 × 10^9^/L, hemoglobin 102 g/L, platelets 166 × 10^9^/L, and neutrophil percentage 97.4%, indicating that the patient was not neutropenic at presentation despite being within 24 h of chemotherapy. C-reactive protein was 16.89 mg/L. Abdominal CT revealed significant gastric retention, small bowel fluid and fecal accumulation, rectal wall thickening with perirectal stranding, hepatic low-density lesions, and perirenal fat stranding (Figure 1). The initial impression included gastrointestinal infection, supraventricular tachycardia, gastric retention, and possible hepatic metastasis.

Supportive management included oxygen therapy, continuous monitoring, intravenous cefoperazone-sulbactam, pantoprazole, and electrolyte glucose solution. Soap water enema was attempted but failed to evacuate stool. A nasogastric tube was placed for gastrointestinal decompression, yielding thick gastric contents. After 3 h of observation in the emergency department, the patient developed shallow breathing, altered consciousness, and gaze fixation. He was immediately transferred to the resuscitation area.

At that point, vital signs deteriorated: blood pressure was unmeasurable, heart rate 154 bpm, respiratory rate 18 breaths/min, and oxygen saturation was undetectable. Arterial blood gas analysis revealed profound metabolic acidosis (pH 6.811), hypercapnia (PaCO₂ 56.5 mmHg), hypoxemia (PaO₂ 71.4 mmHg), bicarbonate 6.9 mmol/L, base excess −24.5 mmol/L, and critically elevated lactate (20.0 mmol/L). The preliminary diagnosis included lactic acidosis, septic shock, supraventricular tachycardia, gastric retention, and renal insufficiency. The patient was declared critically ill, intubated, and placed on mechanical ventilation. Emergency resuscitation included intravenous sodium bicarbonate and isotonic saline. He was subsequently admitted to the intensive care unit for further management.

Upon ICU admission, the patient was febrile (39.3 °C), tachycardic (HR 142 bpm), and normotensive (BP 115/56 mmHg). Repeat laboratory testing—performed approximately eight hours after the initial emergency department evaluation—showed an abrupt decline in WBC to 1.34 × 10^9^/L with neutrophils 88.6%, a pattern consistent with acute deterioration during septic shock rather than the expected chemotherapy related nadir (typically 7–14 days post treatment). Over the subsequent days, bone marrow suppression progressed, reaching a nadir WBC of 0.21 × 10^9^/L on hospital Day 6, before gradually recovering under G-CSF support. Thrombocytopenia (PLT 32 × 10^9^/L), and markedly elevated procalcitonin (>10 ng/mL) were also noted, consistent with severe sepsis. Arterial blood gas confirmed profound metabolic acidosis and hyperlactatemia. The patient remained intubated for respiratory failure and was admitted with a working diagnosis of septic shock and suspected abdominal compartment syndrome (ACS).

In the initial 48 h, the patient required escalating doses of norepinephrine and metaraminol to maintain perfusion. Continuous renal replacement therapy (CRRT) was initiated for severe metabolic acidosis and oliguria. Intra-abdominal pressure (IAP) monitoring confirmed sustained elevations, peaking at 28 mmHg, fulfilling diagnostic criteria for ACS. Gastrointestinal decompression was achieved via nasogastric suction, rectal tube placement, and endoscopic small-bowel drainage. Blood cultures grew ESBL-producing *Escherichia coli*, prompting escalation to meropenem. Fluid resuscitation was guided by PiCCO-derived hemodynamic parameters to balance perfusion with abdominal pressure control.

Over the next several days, the patient developed septic cardiomyopathy with a left ventricular ejection fraction (LVEF) of 27%, cholestatic liver dysfunction (TBIL peaked at 73.5 μmol/L), and coagulopathy (APTT prolonged to 92.5 s). Echocardiography confirmed global hypokinesis. CRRT continued to support renal function and facilitate negative fluid balance. Immunomodulatory therapy included intravenous immunoglobulin, thymosin alpha, and granulocyte colony-stimulating factor (G-CSF).

Hematologic parameters deteriorated sharply in the early ICU phase: WBC dropped to 0.27 × 10^9^/L and PLT to 5 × 10^9^/L by ICU Day 2, requiring repeated transfusions of platelet concentrates and packed red blood cells. Hemoglobin levels fluctuated between 59–65 g/L. Procalcitonin levels surged to >200 ng/mL before gradually declining with antimicrobial therapy and source control. BNP peaked at 2912 pg/mL, reflecting septic myocardial strain, and later decreased in parallel with cardiac recovery.

**Figure 2:**
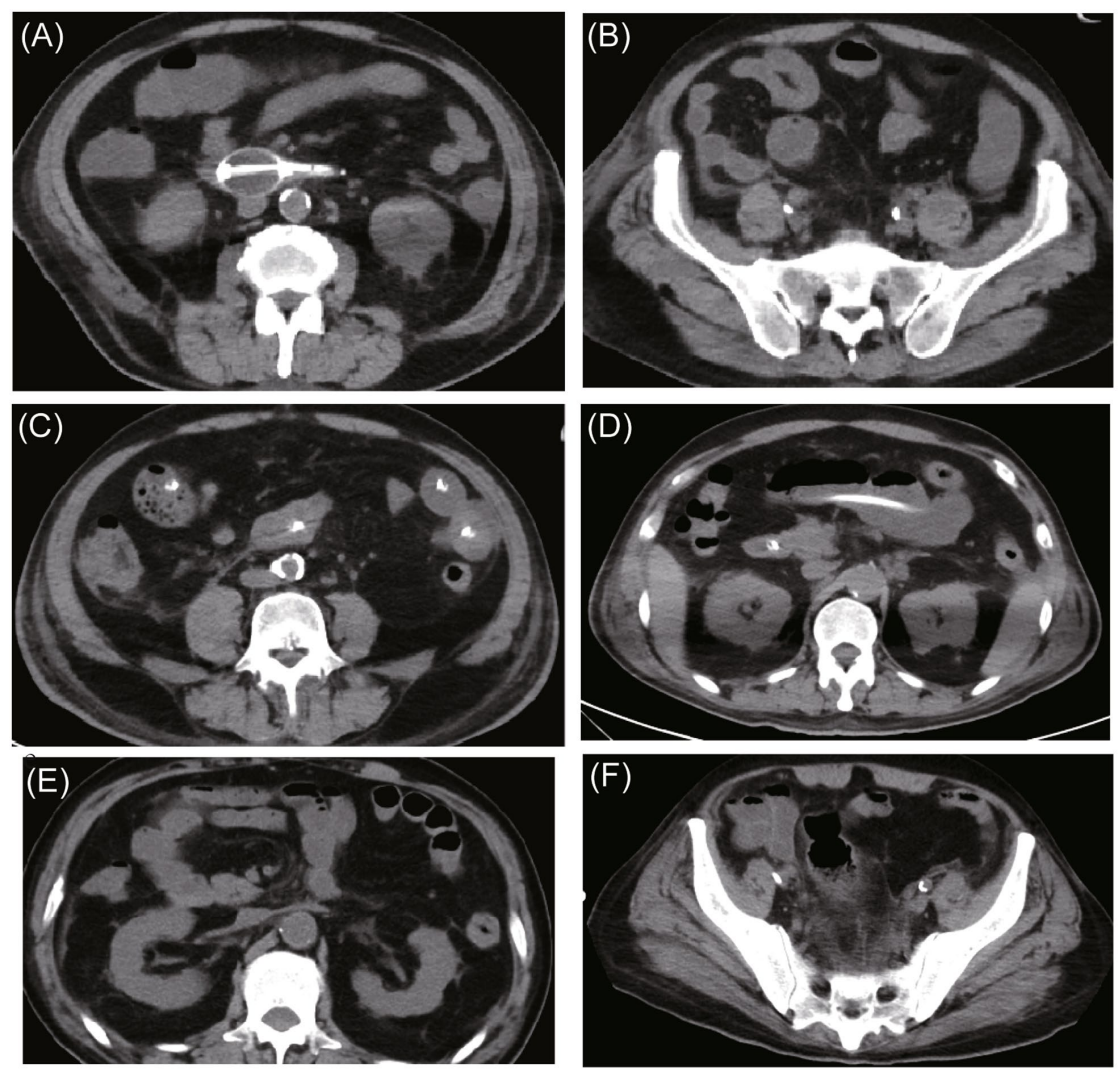
Serial abdomen and pelvic CT scans demonstrating the evolution of intestinal edema and fluid accumulation. **(A,B)** On hospital day 3, thickening of the perirenal fascia are observed. The colon and segments of the small intestine show wall thickening and edema with intraluminal fluid accumulation. A nasogastric decompression tube is in place, draining large volumes of gas and yellowish fluid. **(C,D)** On hospital day 11, post-decompression changes are evident, with partial resolution of small-bowel wall edema. Residual gaseous and fluid distension of the small intestine is present, along with colonic and rectal dilatation containing both gas and fecal material. **(E,F)** On hospital day 25, marked improvement in small-bowel gaseous and fluid distension is seen. However, increased fat stranding around the sigmoid colon persists, suggestive of residual inflammatory changes.

Serial imaging showed progressive resolution of bowel wall edema and intra-abdominal fluid accumulation (Figure 2). IAP decreased to 15 mmHg by ICU Day 6, and organ function began to recover. By ICU Day 9, blood cultures had turned negative, and inflammatory markers showed a marked decline (procalcitonin decreased to 13 ng/mL). By ICU Day 10, WBC had recovered to normal levels, and platelets gradually improved thereafter, consistent with bone marrow recovery under G-CSF support.

The patient was successfully extubated on ICU Day 17 following spontaneous breathing trials and transferred to the general ward on day 19. He demonstrated progressive recovery of bowel function, tolerated enteral nutrition without recurrence of diarrhea, and showed steady hematologic improvement. Sputum cultures later identified *Pseudomonas aeruginosa*, which was effectively managed with targeted antimicrobial therapy and enhanced airway hygiene.

After a total hospitalization of 33 days, the patient was discharged in stable condition. At a three-month follow-up, he reported normal bowel habits, resumed a soft diet without intolerance, and showed no signs of gastrointestinal dysmotility or mucosal dysfunction, indicating sustained recovery of gut integrity. The timeline of key events and clinical parameters for this case is summarized in Table 1.

**Table 1:** Timeline of key events and parameters.

Hospital day	Events/interventions	Infection & antimicrobials	IAP/organ dysfunction	Notable labs/imaging
0	ED: SVT; intubation; vasopressors; NG decompression; ICU admission; PiCCO + CRRT started	Cefoperazone-sulbactam	Severe acidosis; AKI; ARF; shock	pH 6.81, lactate 20 mmol/L; Cr 222 μmol/L; CT: massive GI fluid retention; hepatic low-density lesions
1	Small-bowel decompression tube; continuous IAP monitoring	Escalation per severe sepsis	IAP 28 mmHg; ACS; ongoing shock	PCT >200 ng/mL; BNP 2912 pg/mL; echo planned
2	Hemodynamic optimization via PiCCO; blood/plasma products	Targeted per cultures	Septic cardiomyopathy	Echo LVEF 27%; APTT 92.5 s; WBC 0.71; PLT 5
3–4	Immunomodulation (IVIG, thymosin α); decompression ongoing	Blood cultures: ESBL *E. coli*	IAP down-trending	CT: colonic/small-bowel wall edema and fluid; PCT >200 ng/mL
5–7	De-resuscitation; intermittent CRRT; EN planning	Escalation to meropenem; cultures clearing	IAP ~15 mmHg; organ function recovering	PCT 181 → 73 ng/mL; Cr ~134 → 140 μmol/L; TBil 57 μmol/L; BNP 653 pg/mL
8	Airway care intensified	Sputum: *P. aeruginosa*	Stable hemodynamics	TBil 73.5 μmol/L; WBC 6.1; PLT 10
9–11	CRRT tapered; microbiome restoration (rifaximin, probiotics, oral vancomycin)	—	Improved bowel sounds	PCT 25 → 13 ng/mL; enteral nutrition initiated
12–16	Weaning to low support; SBT; extubation to HFNC	De-escalation of antibiotics	Stable IAP and gas exchange	Cough peak flow 100 L/min; CXR improving
17–19	Rehab; transfer to ward	—	—	WBC 16.7; PLT 97; BNP 426 pg/mL

AKI, acute kidney injury; APTT, activated partial thromboplastin time; ARF, acute respiratory failure; BNP, B-type natriuretic peptide; CXR, chest X-ray; CRRT, continuous renal replacement therapy; CT, computed tomography; EN, enteral nutrition; G-CSF, granulocyte colony-stimulating factor; HFNC, high-flow nasal cannula; IAP, intra-abdominal pressure; ICU, intensive care unit; IVIG, intravenous immunoglobulin; LVEF, left ventricular ejection fraction; NG, nasogastric; PiCCO, pulse contour cardiac output; PLT (×10^9^/L), platelet count; PCT, procalcitonin; PRBCs, packed red blood cells; SBT, spontaneous breathing trial; SIRS, systemic inflammatory response syndrome; TBil, total bilirubin; WBC (×10^9^/L): white blood cell count.

## Discussion

This case exemplifies a rare and clinically consequential progression from chemotherapy-induced gastrointestinal mucositis to ACS—a complication seldom documented in oncology. Chemotherapy-induced mucositis results from epithelial apoptosis, stem cell depletion, microbiome disruption, and cytokine-mediated amplification, creating a vulnerable mucosal barrier prone to bacterial translocation. The sequence began with platinum-etoposide-induced epithelial injury, which disrupted mucosal integrity and compromised the gut barrier. In the setting of neutropenia, this facilitated bacterial translocation and systemic infection. A laxative, administered to address chemotherapy-related dysmotility, likely worsened mucosal disruption, triggering profuse diarrhea and accelerating enterogenic sepsis. Blood cultures confirmed ESBL-producing *Escherichia coli*, implicating the gastrointestinal tract as the infectious source known vulnerability in immunocompromised hosts.

As sepsis progressed, a pronounced inflammatory response ensued, marked by elevated TNF-α, IL-6, and IL-1β. These cytokines increased vascular permeability and drove fluid extravasation into the peritoneal cavity. The resulting capillary leak, compounded by paralytic ileus and impaired abdominal wall compliance, led to rapid intra-abdominal fluid accumulation. Concurrently, aggressive fluid resuscitation—necessary for hemodynamic stabilization—further elevated IAP. In this patient, IAP reached 28 mmHg, accompanied by acute kidney injury, hypoxemia, and circulatory collapse, meeting the diagnostic criteria for ACS as defined by the World Society of the Abdominal Compartment Syndrome (1). This case underscores the complex interplay between systemic inflammation, fluid dynamics, and abdominal physiology in immunocompromised oncology patients.

Although ACS has been described in oncologic contexts, existing reports are limited and primarily involve intraoperative scenarios. For example, Di Giorgio et al. described ACS in a colorectal cancer patient undergoing hyperthermic intraperitoneal chemotherapy (HIPEC), with fluid accumulation during cytoreductive surgery as the precipitating factor (8). This case occurred in surgical oncology settings and involved direct intraperitoneal chemotherapy exposure, differing fundamentally from the present case in etiology, timing, and management approach.

To our knowledge, no prior case report has documented ACS developing in a lung cancer patient as a downstream complication of systemic chemotherapy-induced mucosal injury, without surgical intervention or HIPEC exposure, and successfully managed through a fully nonoperative strategy. This distinction underscores the novelty of our case and highlights a previously underrecognized pathway from chemotherapy-induced gastrointestinal toxicity to life-threatening intra-abdominal hypertension.

Management of ACS in this case adhered to contemporary guidelines favoring nonoperative strategies (9, 10). Gastrointestinal decompression via nasogastric suction, rectal tube placement, and endoscopic small-bowel drainage was initiated early, alongside continuous intra-abdominal pressure monitoring. Fluid resuscitation was guided by PiCCO-derived hemodynamic parameters, allowing for individualized volume management while avoiding further elevation of IAP (11). Continuous renal replacement therapy (CRRT) played a pivotal role in correcting severe metabolic acidosis, supporting renal function, and facilitating negative fluid balance (12). Although the role of CRRT in cytokine clearance remains debated, its early application in this case coincided with clinical improvement and a steady decline in IAP.

The development of septic cardiomyopathy added complexity to the clinical course. Echocardiography revealed a marked reduction in left ventricular ejection fraction (LVEF 27%), which gradually improved with infection control and hemodynamic optimization. This reversible myocardial dysfunction is increasingly recognized in severe sepsis and underscores the importance of cardiac monitoring in critically ill oncology patients (13). The patient’s recovery coincided with the use of immunomodulatory therapies, including intravenous immunoglobulin and thymosin alpha. While these agents were administered as part of a comprehensive supportive strategy, current high-quality evidence does not demonstrate a consistent survival benefit for thymosin alpha in septic patients. A recent multicenter, placebo-controlled randomized trial found no significant improvement in 28-day mortality, suggesting that its role remains investigational (14). Nonetheless, in this individual case, the timing of immunomodulatory therapy paralleled clinical improvement, though causality cannot be established. Additionally, targeted microbiome restoration—using rifaximin, oral vancomycin, and probiotics—was employed to address severe dysbiosis and support gastrointestinal recovery. These interventions were guided by stool culture findings and clinical response, aligning with emerging strategies aimed at restoring gut barrier function in immunocompromised hosts (15). Severe dysbiosis, evidenced by stool cultures dominated by Gram-positive cocci, was addressed with rifaximin, oral vancomycin, and probiotics, contributing to the restoration of gut integrity and function. Probiotics were initiated only after neutrophil recovery, as their use is contraindicated during profound neutropenia due to the risk of probiotic-associated bacteremia or fungemia (16, 17). Immunocompromised patients, including those with chemotherapy-induced neutropenia, have been shown to experience higher rates of bloodstream infections when exposed to probiotic organisms.

This case underscores the importance of early recognition of ACS in immunocompromised patients presenting with abdominal symptoms and septic physiology. Early recognition relies on identifying progressive abdominal distension, worsening tachycardia, new or escalating ventilatory requirements, oliguria, rising lactate levels, and otherwise unexplained metabolic acidosis—features that may precede overt organ dysfunction in high-risk oncology patients. Oncology patients with chemotherapy-induced mucositis and neutropenia are particularly vulnerable to enterogenic sepsis and fluid shifts that predispose to IAH. Continuous IAP monitoring should be considered in such high-risk individuals, especially when abdominal distension, ileus, or escalating ventilatory requirements are present (10). Timely decompression and fluid stewardship are essential to prevent progression to ACS.

The successful reversal of ACS without surgical decompression in this patient highlights the effectiveness of a structured, multidisciplinary approach. Contemporary consensus supports a tiered management strategy (10)—optimizing abdominal wall compliance, evacuating intraluminal contents, managing fluid collections, and tailoring fluid therapy—and early communication with surgical teams is advised when IAP remains refractory despite maximal medical therapy. In this case, profound pancytopenia, septic shock, and the absence of a surgically correctable lesion precluded operative intervention, and guideline-directed nonoperative management was therefore pursued. From a broader perspective, this experience contributes to the evolving understanding of ACS in non-surgical populations, reinforces the need for heightened vigilance in oncology patients, and supports the selective integration of IAP monitoring into routine ICU practice. It also illustrates the complex interplay among chemotherapy, mucosal injury, microbiome disruption, and systemic inflammation—a pathophysiological dynamic of increasing relevance in modern cancer care.

In conclusion, this case exemplifies how chemotherapy-induced mucosal barrier injury can rapidly escalate into life-threatening ACS through a cascade of enterogenic sepsis, systemic inflammation, and fluid overload. Early recognition of evolving intra-abdominal hypertension is particularly critical in these high-risk oncology patients, as delayed identification may allow progression to full abdominal compartment syndrome. This case highlights the importance of early IAP monitoring, individualized fluid management, and nonoperative decompression strategies in high-risk oncology patients. Surgical decompression is rarely feasible in pancytopenic or hemodynamically unstable patients, and guideline-directed conservative management should be prioritized unless refractory IAP persists despite maximal medical therapy. Multidisciplinary, protocolized care anchored in contemporary guidelines is essential for improving outcomes in this vulnerable population.

## Patient perspective

The patient reported that after discharge and during follow-up, he felt significant improvement in quality of life, appreciated the intensive care received, and expressed satisfaction with the recovery of bowel function and overall health.

## Take-home messages

- In immunocompromised oncology patients, chemotherapy-induced mucosal barrier injury can rapidly escalate to enterogenic sepsis, IAH, and ACS.
- Continuous IAP monitoring, early GI decompression, hemodynamic optimization with fluid stewardship, targeted antimicrobials, and timely CRRT can reverse secondary ACS without laparotomy in selected patients.
- Multidisciplinary, protocolized care anchored to ACS and contemporary sepsis guidance is pivotal for survival.

## Data Availability

The datasets presented in this article are not readily available because of ethical and privacy restrictions. Requests to access the datasets should be directed to the corresponding author.
